# From Biomarkers to Behavior: Mapping the Neuroimmune Web of Pain, Mood, and Memory

**DOI:** 10.3390/biomedicines13092226

**Published:** 2025-09-10

**Authors:** Masaru Tanaka, Simone Battaglia

**Affiliations:** 1 Danube Neuroscience Research Laboratory, HUN-REN-SZTE Neuroscience Research Group, Hungarian Research Network, University of Szeged (HUN-REN-SZTE), Tisza Lajos krt. 113, H-6725 Szeged, Hungary; 2 Center for Studies and Research in Cognitive Neuroscience, Department of Psychology “Renzo Canestrari”, Cesena Campus, Alma Mater Studiorum, University of Bologna, 47521 Bologna, Italy; simone.battaglia@unibo.it; 3 Department of Psychology, University of Turin, 10124 Turin, Italy

## 1. Setting the Stage: Mood, Memory and Pain in Clinical Convergence

Mounting evidence situates mood disturbance, memory decline, and chronic pain within a single neuro-immune conversation [1,2]. Cytokines, glial activation, and hypothalamic-pituitary-adrenal signals braid nociceptive and limbic circuits, so a flare in one domain can echo as affective dysregulation or cognitive drift [3,4,5]. Yet biology tells only half the story; social support, fear-avoidance beliefs, and lifestyle patterns modulate this signaling milieu, amplifying or dampening vulnerability [6,7]. Multimodal neuroimaging and plasma proteomics now reveal convergent fingerprints across depression, anxiety, dementia, and pain, hinting at transdiagnostic biomarkers and inviting predictive models that respect context [1,3,8,9,10]. Appreciating this intricate biopsychosocial weave is the first step toward interventions that target both the molecule and the milieu [11,12,13].

Despite rapid expansion of multimodal datasets across depression, anxiety, dementia, and chronic pain, robust transdiagnostic biomarkers remain elusive [14,15]. Meta-analytic neuroimaging reveals both shared and disorder-specific network alterations, while inflammatory and self-referential markers fragment across symptom clusters, underscoring heterogeneity that defeats single-signature diagnostics [8,16,17,18,19]. Clinical translation falters further when biomarker readouts shift with body mass, activity, sleep, medication exposure, and broader lifestyle ecologies; in some cohorts, sickness behavior outperforms cytokines for explaining psychological comorbidity [14,15,20,21]. These inconsistencies demand predictive modeling pipelines that integrate dimensional symptoms, multimodal biology, and environmental context to support individualized risk stratification and adaptive care pathways [15,16,22,23,24]. Without such integrative frameworks, precision interventions will continue to lag behind need in practice [14,15,25,26,27].

This editorial pursues four interconnected goals. First, we integrate preclinical and clinical evidence that maps convergent neuro-immune circuitry linking affective, cognitive, and nociceptive phenotypes. Second, we appraise the most promising, yet still provisional, transdiagnostic biomarkers—from network-level signatures to inflammatory proteomes—assessing their reproducibility and translational readiness. Third, we interrogate how lifestyle and environmental exposures, such as diet, physical activity, and psychosocial enrichment, recalibrate these pathways and modulate symptom trajectories. Finally, we outline robust predictive-modeling architectures that fuse multimodal biology with contextual data, ultimately offering a roadmap toward individually tailored prevention and treatment strategies for co-occurring mood, memory, and pain disorders.

## 2. Where the Gaps Were

Despite prolific datasets, the biological choreography linking affective dysregulation, mnemonic decline, and nociception remains speculative [28,29,30,31,32]. Neuro-immune signals correlate with symptom clusters, yet causal arrows are blurred by cross-sectional snapshots that average out temporal dynamics [28,33,34]. Most human studies still lean on single magnetic resonance imaging (MRI) scans and one-off cytokine panels, analytical conveniences that miss diurnal swings in network excitability or immune tone [28,33,34,35,36]. Consequently, conflicting associations proliferate and mechanistic narratives stall [28,30,33,37,38]. Progress will require multi-scale frameworks in which cell-type gene expression, circuit oscillations, and whole-brain connectomes are integrated within longitudinal designs, permitting inference on how perturbations ripple upward and downward across the neuro-immune hierarchy [28,30,34,39,40].

Longitudinal knowledge is perilously thin. Outside a handful of decade-long registries, few cohorts follow patients as mood symptoms, cognitive drift, and pain wax and wane across treatment cycles; without these trajectories, models cannot learn temporal signatures of risk or recovery [41,42,43]. At the same time, granular information that wearables, smartphones, and in-home sensors could supply remains largely untapped, leaving laboratory snapshots to stand in for lived experience [44,45,46]. When candidate biomarkers do emerge, translation stalls within siloed pipelines: discovery groups rarely collaborate with device engineers, regulators, frontline clinicians, and health-system decision-makers, so results languish in preclinical vaults. These fissures collectively stifle predictive algorithms, thwart stratified trials, and delay the timely delivery of precision care [44,47,48].

Five deficits frame the road ahead: first, a lack of mechanistic alignment linking molecular signals to whole-brain networks [49,50,51]; second, the absence of large, harmonized longitudinal cohorts to chart symptom trajectories [50,52,53]; third, biomarker panels that ignore contextual nuance and remain unstandardized [50,53,54]; fourth, predictive algorithms seldom stress-tested across diverse populations [49,50,52]; and fifth, fragile pipelines that fail to convert discoveries into patient-centered tools [49,52,54] (Table 1).

## 3. Snapshot of the Ten Papers

### 3.1. Chronic Pain and Mechanisms

Zhang and colleagues uncover a unidirectional molecular cascade where spinal neurotrophic tyrosine kinase receptor type 1 (NTRK1) drives insulin-like growth factor II (IGF2) expression, fueling endoplasmic reticulum (ER) stress and sustaining chronic postsurgical pain [55]. Using the skin/muscle incision–retraction (SMIR) rat model, they show that blocking tropomyosin receptor kinase A (TrkA) or silencing IGF2 breaks this pathogenic loop. Their findings highlight a novel, non-opioid target for precision pain therapy. In this prospective real-world study, Della Vecchia and colleagues reveal that anti-calcitonin gene-related peptide/receptor (CGRP/R) monoclonal antibodies do more than just ease migraine pain [56]. They also alleviate depression, anxiety, fatigue, and allodynia in treatment-resistant patients. Interestingly, even non-responders showed some psychiatric improvement, hinting at deeper neuroaffective effects worth exploring. (Table 2).

### 3.2. Comorbidity and Cognitive Decline

This study brings fresh light to an urgent issue: how gum health might reflect brain health. Using data from the PerioMind Colombia Cohort, the authors show that specific periodontal indices—like gingival redness and pocket depth—closely align with mild cognitive impairment in older adults [57]. Their models suggest dentists could become frontline detectors of cognitive decline. This compelling study explores how comorbidities influence the transition from mild cognitive impairment to dementia. By clustering patients based on their comorbidity profiles, the authors uncover striking risk patterns—diabetes and obesity sharply accelerate decline, while traumatic brain injury shows surprisingly low progression [58]. It is a sharp call to personalize dementia risk management. This study offers a fresh lens on predicting dementia. Tracking 132 individuals with mild neurocognitive disorder over two years, the authors crafted a model that nails an 83.7% accuracy rate [59]. As for risk, it rises with alcohol and a higher body mass index (BMI). In terms of protection, female sex, strong praxis, and surprisingly, higher scores on the geriatric depression scale (GDS) prevail. This study uses simple inputs to obtain powerful foresight.

### 3.3. Neuro-Immune Stress Circuitry

Smagin and colleagues dive deep into the biological aftermath of social stress [60]. Using a robust mouse model, they reveal striking shifts in blood plasma proteins linked to depression and neurodegeneration. What stands out? A telltale signature of amyloid and apolipoproteins—mirroring patterns seen in Alzheimer’s. A compelling read. This elegant review explores how neuroinflammation, oxidative stress, and mitochondrial dysfunction converge in major depression—and how phytochemicals might disrupt that vicious cycle [61]. Drawing from both bench and bedside, the authors highlight natural compounds like curcumin and flavonoids as promising multitarget tools for mood modulation, with fewer side effects and broader neuroprotective impact than traditional drugs [65].

### 3.4. Lifestyle and Biopsychosocial Angles

In this timely study, the authors reveal how being inactive physically can turn up the volume on the connection between anxiety, depression, and neck pain in breast cancer survivors. Surprisingly, this link does not hold for low back pain [62]. Their message is clear: movement matters—and not just for the body, but the mind too. This timely and thorough review dives deep into the tangled web of chronic orofacial pain [63]. It challenges outdated diagnostic norms and champions a biopsychosocial lens—merging the International Classification of Diseases, Eleventh Revision (ICD-11); the International Classification of Headache Disorders, Third Edition (ICHD-3); the International Classification of Orofacial Pain (ICOP); and the Diagnostic and Statistical Manual of Mental Disorders, Fifth Edition (DSM-5) criteria. This way, the authors spark a necessary rethinking of classification and care, urging a unified, patient-centered approach.

### 3.5. Neurodevelopment and Novel Targets

Samra and colleagues thrust the carotenoid astaxanthin into the autism spotlight [60]. In valproic acid (VPA)-exposed rats, the pigment steered the renegade renin–angiotensin axis toward its protective arm, muted Notch signaling and nuclear factor kappa B (NF-κB) activation, untangled microtubule-associated protein tau, softened gliosis, and, crucially, coaxed the pups back into sociability. Biology and behavior clicked in one tidy preclinical arc.

## 4. Bridges Built: How These Studies Advance the Field

By deploying the SMIR rat paradigm, investigators traced a spinal NTRK1-triggered cascade that amplifies IGF2 expression and culminates in maladaptive ER stress, a trio now validated as driver of postsurgical pain [55]. Parallel real-world trials with anti-CGRP/R monoclonals show that targeting sensory peptides simultaneously softens migraine, depression, anxiety, and mechanical allodynia [56]. Together these studies fuse molecular neurobiology with bedside pain management, sketching an immediate roadmap for non-opioid interventions and broader psychiatric benefit in chronic headache populations [66,67,68].

Periodontal indices now function as surrogate biomarkers of neural integrity, with gingival redness, pocket depth, and alveolar loss tracking early cognitive slippage [57]. Clustering comorbidities such as diabetes, obesity, and cardiovascular burden sharpens dementia risk stratification by exposing synergistic rather than additive trajectories [58]. This cross-pollination between dentistry, neurology, and geriatric psychiatry recasts the dental clinic as a cognitive sentinel, enabling nuanced risk profiles that drive personalized interventions and feed next-generation prediction algorithms [57,58,59].

Plasma proteomic profiling after controlled social stress uncovers a signature that bridges cytokine flux with neurodegenerative cascades [60,69]. Parallel animal and cell studies show curcumin, quercetin, and related phytochemicals dampening microglial activation and improving mitochondrial function, positioning natural compounds as innovative neuroimmune modulators [61,70]. Integrating immunologic analytics with psychiatric phenotyping clarifies how inflammatory and mitochondrial crosstalk drives depressive symptoms [71]. These converging insights nominate biomarkers for depression and prodromal Alzheimer’s disease while advancing low-toxicity phytochemical therapeutics over conventional antidepressants [72,73,74].

By isolating physical inactivity within post-cancer cohorts, targeted analyses compute novel multivariate paths, exposing sedentarism as a catalyst of the anxiety-depression-pain triad in breast cancer survivors [62,75,76]. Psychology, oncology, and rehabilitation converge, refreshing the biopsychosocial paradigm [77,78,79]. Such transdisciplinary clarity reorients clinical priorities. Evidence thus advocates early, personalized activity prescriptions to blunt cascading affective and nociceptive burdens and to integrate mental-health prevention into routine oncology pain care [75,77,78,80].

By fusing ICD-11, ICHD-3, ICOP, and DSM-5 within a biopsychosocial scaffold, these studies recalibrate neurodevelopmental disorder nosology, exposing phenotypes that transcend categorical silos [63,81,82]. Such conceptual innovation invites neurologists, psychiatrists, dentists, and developmental scientists into a shared space, aligning biomarkers with behavioral heuristics and unveiling novel therapeutic targets [81,83]. Clinically, patient-centered algorithms and interdisciplinary guidelines emerge, accelerating therapeutics from bench to bedside [81,82,84].

## 5. Still Unpaved Roads: Future Research Directions

### 5.1. Multi-Omics Mega-Cohorts

Big data only matters if it stays in step with biology [85,86,87]. The next wave should launch mega-cohorts that blend single-cell transcriptomics, proteomics, metabolomics, and connectomics with repeated neuroimaging and biosamples every three to six months [85,88,89,90]. Such “rolling deep phenotypes” will let us watch how a flare of neuropathic pain, a dip in mood, or a slip in recall leaves footprints from microglia to default-mode hubs [91,92,93]. Harmonized pipelines and open cloud workspaces will finally allow algorithms to trace directional signals, validating the mechanistic hypotheses flagged in the gaps above and building a living atlas that evolves with each new participant—and across diverse ancestries, socioeconomic contexts, and life stages, capturing latent resilience alongside vulnerability [94,95].

### 5.2. Longitudinal Pain-Mood Tracking and Lifestyle-Targeted Randomized Controlled Trials (RCTs)

Pair those omics streams with real-world longitudinal tracking. Smartphone diaries, passive gait sensors, and cross-platform ecological momentary assessment (EMA) can capture minute-to-minute swings in pain intensity, affect, sleep, and cognitive fog [96,97,98,99]. By feeding those trajectories into adaptive, lifestyle-targeted randomized controlled trials (RCTs)—think exercise-timing, personalized nutrition, light therapy, or mindfulness-dose algorithms—we can test causal levers in near real time [98,100]. Each participant becomes a micro-trial, and aggregated n-of-1 readouts will reveal who benefits, when, and why [100]. This approach does more than shrink sample sizes; it closes the discovery-translation loop, letting mechanistic insights instantly inform behavioral prescriptions delivered at the wrist [98,100].

### 5.3. Cross-Talking Neuro-Immune Combination Therapies

The final gap is therapeutic: neuro-immune dialog rarely respects disciplinary borders. Hybrid interventions that co-modulate cytokine tone and neural circuit excitability—such as low-dose interleukin (IL)-1 antagonists paired with transcranial alternating current, vagus-paced breathing, or gut-microbiota modulators—could rewrite maladaptive set points [101,102,103]. We need factorial trials that cross these modalities, mapped against the multi-omics atlas, to pinpoint synergistic nodes [104,105,106]. A regulatory sandbox shared by neuroscientists, immunologists, and digital-health engineers would speed iteration, while federated analytics guard privacy. Success would move us from symptom silos to network rewiring, turning the still-unpaved road into an integrated highway toward precision recovery [107].

## 6. Take-Home Message

Together, these ten papers narrow down the critical mechanistic and translational gaps—further developing our understanding of how mood, memory, and pain braid through neuro-immune circuitry—while simultaneously prying open fresh investigative frontiers. From spinal NTRK1-IGF2 loops that propel postsurgical hyperalgesia to gingival biomarkers that foreshadow cognitive drift, this collection delivers concrete answers yet sparks new questions about bidirectional brain–body crosstalk. Proteomic signatures, connectomic shifts, and wearable-captured behaviors now coalesce into a reproducible risk compass, steering clinicians toward earlier, kinder interventions. Lifestyle, comorbidity, and social context emerge not as confounders but as tunable levers, suggesting that a step counter or food log can be as informative as a positron emission tomography (PET) scan. The horizon beckons with multi-omics cohorts tethered to smartphone phenotyping, adaptive n-of-1 trials that turn each patient into their own laboratory, and combination neuro-immune therapies poised to retune both cytokine tone and cortical rhythm. With the stage now set, the next wave of precision relief is already cresting.

## Figures and Tables

**Table 1:** Key research deficits in translational neuropsychiatry. Five major gaps hinder progress in precision neuropsychiatric care, spanning mechanistic integration, longitudinal tracking, biomarker standardization, algorithmic robustness, and translational pipelines.

	Deficit	References
1	Lack of mechanistic alignment linking molecular signals to whole-brain networks	[49,50,51]
2	Absence of large, harmonized longitudinal cohorts to chart symptom trajectories	[50,52,53]
3	Biomarker panels that ignore contextual nuance and remain unstandardized	[50,53,54]
4	Predictive algorithms seldom stress-tested across diverse populations	[49,50,52]
5	Fragile pipelines that fail to convert discoveries into patient-centered tools	[49,52,54]

**Table 2:** Thematic clustering of ten recent papers relevant to mood, memory, and pain convergence. CGRP/R, calcitonin gene-related peptide/receptor; ER, endoplasmic reticulum; IGF2, insulin-like growth factor II; NTRK1, neurotrophic tyrosine kinase receptor type 1.

Thematic Group	Paper Title (Shortened)	References
Chronic Pain and Molecular Mechanism	Unidirectional Crosstalk Between NTRK1 and IGF2 Drives ER Stress in Chronic Pain	[55]
	Impact of Anti-CGRP/R mAbs on Comorbid Symptoms in Resistant Migraine	[56]
Comorbidities and Cognitive Decline	Periodontal Indices as Predictors of Cognitive Decline (PerioMind Cohort)	[57]
	Impact of Comorbid Conditions on Conversion from Mild Cognitive Impairment to Dementia	[58]
	Predictive Models for Progression to Major Neurocognitive Disorder	[59]
Neuroinflammation, Stress, and Depression	Blood Plasma Markers in Mice under Chronic Social Defeat Stress	[60]
	Neuroinflammation and Natural Antidepressants	[61]
Behavioral and Lifestyle Factors in Pain, Depression, and Anxiety	Physical Inactivity Amplifies Anxiety, Depression, and Neck Pain in Breast-Cancer Survivors	[62]
	Advances in Classifying Chronic Orofacial Pain via Biopsychosocial Models	[63]
Neurodevelopmental Disorders and Novel Therapeutic Targets	Yin/Yang Angiotensin System Components in Autism Spectrum Disorder; Astaxanthin as Therapy	[64]

## Abbreviations

The following abbreviations are used in this manuscript:

CGRP/R	Calcitonin gene-related peptide/receptor
DSM-5	Diagnostic and Statistical Manual of Mental Disorders, Fifth Edition
ER	Endoplasmic reticulum
IGF2	insulin-like growth factor II
ICD-11	International Classification of Diseases, Eleventh Revision
ICHD-3	International Classification of Headache Disorders, Third Edition
ICOP	International Classification of Orofacial Pain
NTRK1	Neurotrophic tyrosine kinase receptor type 1
